# Effects of Leisure-Time Physical Activity on Vertebral Dimensions in the Northern Finland Birth Cohort 1966

**DOI:** 10.1038/srep27844

**Published:** 2016-06-10

**Authors:** Petteri Oura, Markus Paananen, Jaakko Niinimäki, Tuija Tammelin, Sauli Herrala, Juha Auvinen, Raija Korpelainen, Juho-Antti Junno, Jaro Karppinen

**Affiliations:** 1 Medical Research Center Oulu, Oulu University Hospital and University of Oulu, Oulu, Finland; 2 Center for Life Course Health Research, Faculty of Medicine, University of Oulu, Oulu, Finland; 3 Research Unit of Medical Imaging, Physics and Technology, Faculty of Medicine, University of Oulu, Oulu, Finland; 4 LIKES—Research Center for Sport and Health Sciences, Jyväskylä, Finland; 5 Department of Sports and Exercise Medicine, Oulu Deaconess Institute, Oulu, Finland; 6 Cancer and Translational Medicine Research Unit, Faculty of Medicine, University of Oulu, Oulu, Finland; 7 Department of Archaeology, Faculty of Humanities, University of Oulu, Oulu, Finland’; 8 Finnish Institute of Occupational Health, Oulu, Finland

## Abstract

Vertebral fractures are a common burden amongst elderly and late middle aged people. Vertebral cross-sectional area (CSA) is a major determinant of vertebral strength and thus associated with vertebral fracture risk. Previous studies suggest that physical activity affects vertebral CSA. We aimed to investigate the relationship between leisure-time physical activity (LTPA) from adolescence to middle age and vertebral dimensions in adulthood. We utilized the Northern Finland Birth Cohort 1966, of which 1188 subjects had records of LTPA at 14, 31 and 46 years, and had undergone lumbar magnetic resonance imaging (MRI) at the mean age of 47 years. Using MRI data, we measured eight dimensions of the L4 vertebra. Socioeconomic status, smoking habits, height and weight were also recorded at 14, 31 and 46 years. We obtained lifetime LTPA (14–46 years of age) trajectories using latent class analysis, which resulted in three categories (active, moderately active, inactive) in both genders. Linear regression analysis was used to analyze the association between LTPA and vertebral CSA with adjustments for vertebral height, BMI, socioeconomic status and smoking. High lifetime LTPA was associated with larger vertebral CSA in women but not men. Further research is needed to investigate the factors behind the observed gender-related differences.

Osteoporosis is a bone deficiency disease with a high global prevalence among aging population and the elderly. It often leads to non-traumatic vertebral fractures with consequent morbidity and has negative effects on the quality of life^1^,^2^. A systematic review suggested that small vertebral size is an independent risk factor for vertebral fractures^3^. Therefore, knowledge on factors which influence vertebral morphology would be beneficial^4^.

Vertebral bone strength is determined by architectural, material and geometrical properties^5^,^6^,^7^,^8^. Causing changes in these factors, age affects vertebral strength to a large extent^8^,^9^,^10^. Men are known to have larger vertebral cross-sectional area (CSA), both absolute and relative to body size, as well as greater compressive strength than women^1^,^11^. Regardless of gender, bone mass reaches its maximum value between the ages of 20 and 30 years, decreasing thereafter^10^. Among males, vertebrae seem to increase in size over life, whereas among women the findings on the age-related increase in size are controversial^6^,^8^,^9^,^12^,^13^. Unlike vertebral size, bone mineral density (BMD), which is a major indicator of vertebral strength^3^, has been found to decrease in both genders throughout age^6^.

Physical activity is known to influence bone through dynamic biomechanical loading, especially when performed frequently on a regular basis^14^. Participation in different sports has been reported to affect bone density, shape and size to a varying extent^14^,^15^,^16^. Regarding long bones, mechanical strain has shown to increase their cross-sectional size and bone mineral density^17^,^18^,^19^,^20^. Mechanical loading of vertebrae has, in addition, been associated with increased vertebral dimensions and bone mass^8^.

Bones respond to loading in a site-specific manner^18^,^21^. It is suggested that the morphological mechanisms behind exercise-induced changes in bone size are 1) an increase in cortical bone thickness, which is prevalent during the growth period, and 2) a decrease in endosteal bone loss, which is prevalent in the ageing population^22^.

According to literature, leisure-time physical activity (LTPA) has for long been proposed to associate with increased strength of the lumbar spine, as well as higher vertebral BMD^23^,^24^. Some recent studies have confirmed these findings^25^,^26^, whereas others have not detected a similar association^27^,^28^. The risk of vertebral fractures decreases in relation with increasing back extensor muscle strength^29^. Many studies have shown a higher incidence of vertebral fractures in women compared to men and the underlying reason for this difference has been suggested to be the more active periosteal bone formation in men^30^. Among women, the incidence of new vertebral fractures clearly increases with age^31^. However, the findings on gender differences in the incidence and risk of vertebral fractures are controversial^1^,^32^,^33^,^34^.

The relationship between lifetime LTPA and vertebral bone size is not thoroughly explored. We have previously discovered a secular decrease in vertebral size from the Medieval times^35^ which may be the result of a temporal decrease in the human physical activity level. However, our other previous studies have not supported this theory, as LTPA level and participation in different sports during late adolescence were not associated with vertebral size at 21 years of age^36^,^37^.

In this study we attempted to clarify the role of lifetime LTPA (14–46 years of age) on vertebral dimensions and thus also on biomechanical strength of vertebrae. We utilized the large population-based Northern Finland Birth Cohort 1966 (NFBC1966) to study the association between LTPA from 14 to 46 years of age and vertebral size at 47 years of age. We hypothesized that high LTPA was associated with increased vertebral CSA.

## Results

### Study sample

A total number of 524 males (44.1%) and 664 females (55.9%) were included in the analyses (Table 1). The mean age of imaging was 46.8 years for men (standard deviation, SD, 0.4 years, range 45.9–47.8 years), and 46.8 years for women (SD 0.4 years, range 45.8–48.0 years). In our sample, 34% of men and 49% of women were within the normal Body mass index (BMI) range, while others were overweight. Most subjects (72% of men and 73% of women) had attended school for 9 to 12 years and had never smoked on a regular basis (52% of men and 61% of women).

### Leisure-time physical activity

In the latent class analysis (LCA), three lifetime LTPA clusters were identified as the most appropriate solution. The clusters were named as “inactive”, “moderately active” and “active”; the distributions of LTPA at 14, 31 and 46 years are shown for each cluster in Table 2. Based on the LCA, 29% of men and 24% of women were classified as active, whilst 29% and 30% were classified as inactive, respectively.

### Vertebral dimensions

The mean vertebral CSA was 13.25 (SD 1.67) cm^2^ among males and 10.57 (SD 1.28) cm^2^ among females in our sample (25.4% higher among males). The measurements of L3 and L4 showed a high correlation (Pearson’s R = 0.870, p < 0.001) in the CSA of these vertebrae and the level of intra-rater reliability was high (intraclass correlation = 0.963). The values of relative measurement error (%) distributed normally around the mean of 0.0 with a standard deviation of 4.9 (n = 400 repeated measurements).

When investigating the association between LTPA and vertebral CSA using linear regression, we found that unadjusted and adjusted analyses provided similar results (Tables 3 and 4). However, the F-test p values supported statistical significance of the adjusted models (p < 0.01 in all models), whereas the unadjusted models all had a p value of >0.05. According to the R^2^ values, the unadjusted models, utilizing only the LTPA variables without adjustments, explained <1% of the variability in the CSA of the subjects. The adjusted models, in turn, explained 5.2–6.6% of the variety.

In the analysis of the association between lifetime LTPA and vertebral CSA (Table 3), those women who belonged to the “active” cluster had 0.34 cm^2^ (3.2%) larger vertebral CSA, compared to those who belonged to the “inactive” cluster (p = 0.012). No statistically significant differences were detected in the CSA between women classified as “inactive” and “moderately active”. Among males, no statistically significant differences were detected.

In the additional analyses regarding LTPA at different time points (Table 4), we detected that those women who were physically active ≥4 times per week at 31 years, had 0.47 cm^2^ (4.5%) larger vertebral CSA than the reference group (p = 0.003). We also detected that those men who were active 2 times per week at 14 years, had smaller CSA compared to the reference group (p = 0.027). No other statistically significant findings were obtained.

## Discussion

In this population-based birth cohort study, our main finding was that a high level of lifetime LTPA from 14 to 46 years of age was associated with larger vertebral CSA in middle-aged women. Our additional analyses showed that the participation frequency of ≥4 times/week at age 31 was associated with larger vertebral CSA in women at 47 years. However, the detected CSA differences were of minor magnitude, and physically active and inactive men had similar vertebral dimensions.

In our previous study, physical activity and participation in different sports were not associated with changes in vertebral size at the age of 21 years^37^. The lack of association between physical activity and vertebral size then may be due to the young age of the study population. In the present study, we wanted to focus on the overall amount of LTPA instead of investigating individual sports. We used an older study population in which all individuals had reached their skeletal maturity and peak bone mass. The sample size we used was rather large, compared to both our previous studies and other research on vertebral dimensions^3^,^36^,^37^. The NFBC1966 study provided us with longitudinal data on the subjects from adolescence until middle-age. As the subjects shared their birth year, the confounding effect of age was minimized.

Several studies have analyzed the dimensions of other lumbar segments, e.g. L3, instead of L4^3^, possibly affecting the comparability of results. Previous literature has described the similarity of vertebrae in terms of dimensions and strength, concluding that the strength prediction of all thoracolumbar vertebrae can be made with high accuracy using measurements from one vertebra^38^. Our comparisons between the dimensions of L3 and L4 confirmed that the size of different lumbar segments is highly linked and so our study is comparable with others, regardless of the vertebra investigated. We decided to focus our interest on the fourth lumbar vertebra (L4), as it is situated in the inferior part of the vertebral column and therefore is amongst those vertebrae that carry the most weight. L5 too is under major strain but it is also an integral part of the lordotic curvature of the lumbar spine^39^,^40^, and therefore was not measured. We acknowledge that due to lumbar lordosis, the location and orientation of also L4 varies between individuals. This may have affected our orientation of the MR slices and thus added to the measurement error.

There are many potential explanations for the observed gender differences. Given that women have smaller vertebrae, both absolute and relative to body size, there might be a higher ability or a greater demand for the vertebrae to enlarge in size. Furthermore, the men in our study sample were somewhat more physically active than women, which may prevent us from detecting a similar effect of LTPA on vertebral size. The ambiguous, inexplicable association between LTPA at a frequency of 2 times/week at age 14 and decreased vertebral CSA in men was not in line with our other results.

Another potential hypothesis is that the female gender may have vertebral compensatory mechanisms, such as periosteal apposition^3^, functioning differently from men. In males, periosteal apposition could be activated at lower LTPA levels, leaving the threshold undetectable; it might thus explain the increase in bone diameter in all men, regardless of LTPA. Interestingly, it has been suggested that androgens increase periosteal apposition, whereas estrogen levels have been shown to decrease it^41^. Intervertebral disc degeneration has also been linked with higher vertebral bone growth tendency^42^ but the degree of disc degeneration was not evaluated by us when reading lumbar MR images.

We are aware that the effect of physical activity on vertebrae may be expressed in other ways than changes in vertebral dimensions which were investigated in this study. The alternative mechanisms might be e.g. cortical shell thickening and/or changes in vertebral BMD. However, our previous study did not find any correlation between LTPA and trabecular bone density parameters in vertebrae^37^.

Our study suggests that lifetime LTPA is positively associated with vertebral size, to a small extent, among women but not men. Further research is needed to confirm our findings and shed light on the factors behind the observed gender-related differences.

## Methods

### Study population

Individuals whose expected date of birth fell between January 1^st^ and December 31^st^ 1966 in Northern Finland (96.3% of all 1966 births, n = 12,058 live births) were included in the prospective NFBC1966 study. Since their mothers’ recruitment during their first visit to the maternity health centers, data have been collected on the subjects’ health, lifestyle and social status. The study was conducted according the Declaration of Helsinki and approved by the Ethical Committee of the Northern Ostrobothnia Hospital District in Oulu, Finland. Cohort members and in youth also their parents provided written informed consent for the study. All personal identity information was encrypted and replaced with identification codes, providing full anonymity for the whole study population. We confirm that the study was carried out in accordance with the approved guidelines.

**Figure 1:**
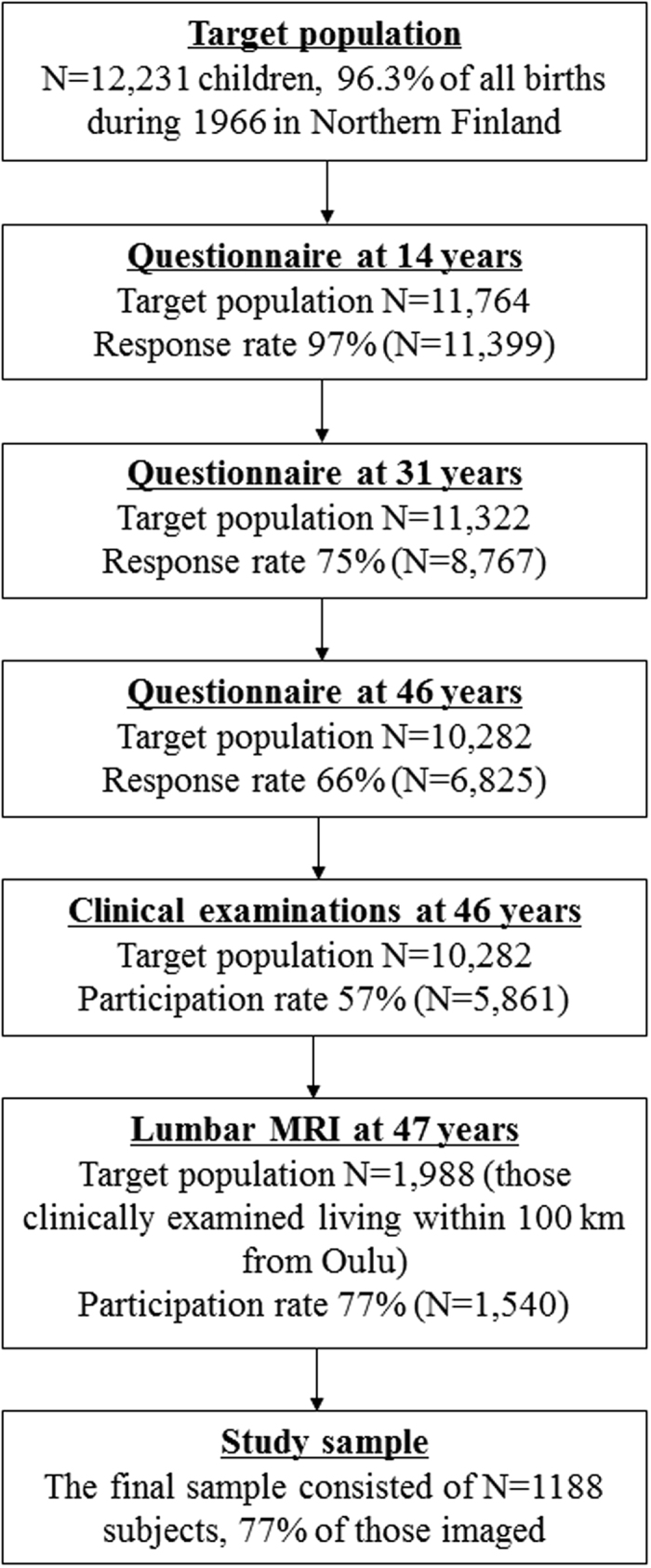
Formation of the study sample.

Postal questionnaires enquiring about the participants’ health status and lifestyle were conducted in 1980, 1997–1998, and 2012–2014, when the participants were aged 14, 31 and 46 years, respectively (Fig. 1). The questionnaires were mailed to all subjects whose addresses were known. The response rate was 97% at age 14 (n = 11,399), 75% at age 31 (n = 8,767), and 66% at age 46 (n = 6,825).

At the age of 46 years, subjects who were living at known addresses in Finland (n = 10,282) were invited to attend clinical examinations (Fig. 1). A total of 5,861 (57%) subjects participated. Of these, we invited all participants living within 100 km of the city of Oulu (n = 1,988) to lumbar magnetic resonance imaging (MRI). Of them, MRI was not performed to 448 subjects due to 1) not showing up, 2) claustrophobia, 3) severe obesity preventing the imaging, or 4) a pacemaker. The final MRI study population consisted of 1540 participants.

After the MR imaging, 352 subjects (23%) were excluded from the study due to 1) inability to measure vertebral dimensions (segmentation error, endplate erosion, severe disc degeneration, spondylodesis, and/or presence of Schmorl’s nodes; n = 147), 2) bone-affecting medication (calcium supplements and/or osteoporosis medication; n = 26), or 3) missing LTPA or covariate data (n = 179). Therefore the final eligible population consisted of 1188 participants.

### Assessment of leisure-time physical activity at 14, 31 and 46 years

Physical activity was self-reported at 14, 31 and 46 years of age^43^. At the age of 14, subjects were asked how often they participated in sports outside school hours with the following alternatives: 1) daily, 2) every other day, 3) twice a week, 4) once a week, 5) every other week, 6) once a month, and 7) generally not at all. The subjects having answered either 5, 6 or 7 were combined into one category (“every other week or less”), as the groups were small. Other categories were kept as-is. At the ages of 31 and 46, subjects were asked how often they participated in brisk physical activity/exercise during their leisure-time. The term ‘brisk’ was defined as physical activity causing at least some sweating and getting out of breath, corresponding to moderate-to-vigorous intensity. The six response alternatives were 1) daily, 2) 4–6 times a week, 3) 2–3 times a week, 4) once a week, 5) 2–3 times a month, and 6) once a month or less often. The subjects having answered either 1 or 2 were combined into one category (“≥4 times/week”), as the number of respondents was small, and other categories were kept as-is.

### Covariates at 46 years

At the age of 46, subjects underwent clinical examinations, where their height and weight were systematically measured by a trained study nurse. Body mass index (BMI) was then calculated for each subject (kg/m^2^). Socioeconomic status was evaluated based on the number of years the subject had attended school for (≤9 years, 9–12 years, >12 years). This was determined by asking: “What is your basic education?” 1) Less than 9 years of ground school, 2) ground school, or 3) matriculation examination. Smoking history and current smoking status were inquired with two questions: 1) “Have you ever smoked cigarettes (yes/no)?” and 2) “Are you currently smoking (yes/no)?” According to these questions, three categories were formed: 1) non-smoker, 2) former smoker, and 3) current smoker.

### Lumbar magnetic resonance imaging

Magnetic resonance imaging scans were performed with a 1.5-T imaging system (Signa HDxt, General Electric, Milwaukee, WI) between 2012 and 2014 when the participants were averagely 47 years old. The imaging sequences followed routine lumbar spine protocol including T2-weighted fast-recovery fast spin-echo (frFSE) images in sagittal (TR/effTE 3500/112 ms, 4 averages, FOV 280 × 280 mm, acquisition matrix 448 × 224, slice thickness 3 mm with 1 mm interslice gap) and transverse planes (TR/effTE 3600/118 ms, 4 averages, FOV 180 × 180 mm, acquisition matrix 256 × 224, slice thickness 4 mm with 1 mm interslice gap).

**Figure 2:**
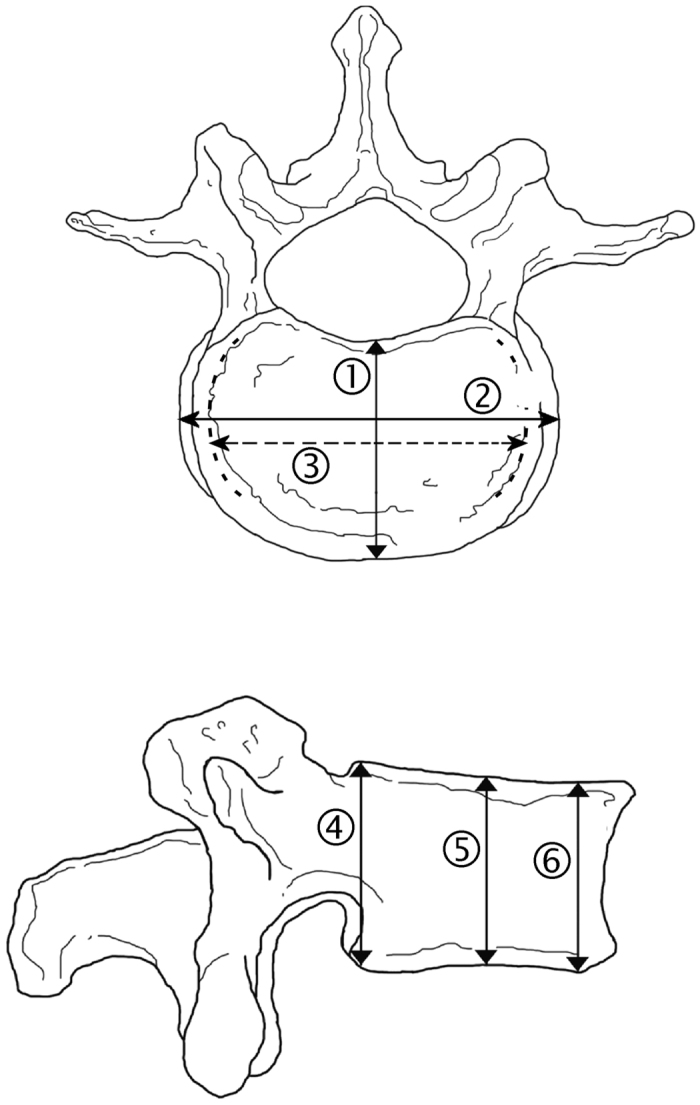
Dimensions measured from the vertebra. Depth, i.e. anteroposterior length, of the vertebra superiorly (measurement 1), halfway (not shown) and inferiorly (not shown); maximum mediolateral width (measurement 2) and minimum mediolateral width (measurement 3) of the vertebra; height of the vertebra posteriorly (measurement 4) and anteriorly (measurement 6), and the minimum height dimension between them (measurement 5).

We measured 8 dimensions from the corpus of the L4 vertebra (Fig. 2) to calculate the axial cross-sectional area and mean height of L4. Vertebral height dimensions (anterior height, posterior height, minimum height) were measured using the sagittal view and the most medial slice that was available. Width dimensions, i.e. minimum mediolateral width and maximum mediolateral width, were measured using the appropriate axial slices that varied between subjects. Typically the minimum width was encountered near the middle part of the vertebra and the maximum width near either the superior or inferior end of the vertebra. Depth dimensions, i.e. anteroposterior dimensions, were measured using axial slices. The superior depth dimension was measured using the most superior appropriate slice just before the intervertebral disc. Correspondingly, the inferior depth dimension was measured using the most inferior slice possible. In order to measure the middle depth dimension we chose the slice that existed halfway between the superior and inferior ends of the vertebra.

CSA values were calculated by using the acknowledged formula CSA = π * a * b, where a = vertebral width/2 and b = vertebral depth/2^44^. The mean of maximum and minimum mediolateral dimensions was used as the width dimension, and the mean of superior, inferior and middle anteroposterior dimensions was used as the depth dimension (Fig. 2). We additionally used vertebral height as a covariate in the analysis. This height dimension was calculated as the mean of anterior, posterior and minimum height dimensions of L4.

All MRI measurements were performed by the same researcher, using the NeaView Radiology software (Neagen Oy, Oulu, Finland) version 2.31, which is collectively in use on clinical workstations in Oulu University Hospital. The measurements were made prior to gathering or analyzing any other data on the subjects. To explore the correlation between dimensions of different vertebrae, we also measured the same dimensions from the L3 vertebra from a subsample (n = 110).

### Intra-rater reliability and measurement error

In order to investigate intra-rater reliability, 50 MR images (equivalent to n = 400 measurements) were randomly selected for a second measuring three weeks after the first one, conducted by the original measurer. Based on the original and repeated measurements, intraclass correlation coefficient was calculated. Additionally, measurement errors were calculated. As the height, width and depth dimension values were of different magnitudes, we considered relative error (%) more informative as opposed to investigating absolute error.

### Statistical analyses

Latent Class Analysis (LCA) was used to obtain clusters, i.e. groups in which the subjects had a similar profile of LTPA during their lifetime (14–46 years of age). In LCA, the number of clusters is increased until the most appropriate model is found^45^,^46^. In this study, the LCA was conducted according to the self-reported frequency of LTPA at 14, 31 and 46 years of age. Models with cluster numbers from one to seven were assessed, and the best-fitting cluster model for these subjects was determined by calculating the Bayesian Information Criterion (BIC), where lower values indicate a better fit. Clinical interpretability of the classification, the conceptual meaningfulness of the models, and the sizes of the subgroups were also assessed while choosing the best model. In order to obtain equal definitions of the LTPA clusters in both men and women, and thus ensure the comparability of results between genders, the clustering was not stratified by gender.

We used linear regression analysis to reveal the association between LTPA and vertebral size. Vertebral size, i.e. the CSA of L4, was regarded as the dependent variable in all analyses, whereas LTPA variables acted as the explanatory variables. As both CSA^12^ and LTPA^47^,^48^ were known to differ between genders, a significant gender interaction was expected and therefore all regression analyses were performed separately for men and women. The models were adjusted for 1) height of the L4 vertebra, continuous variable; 2) BMI at 46 years, continuous variable; 3) socioeconomic status determined by education years, categorical variable; and 4) lifetime smoking status determined at 46 years, categorical variable. These determinants were known to associate with changes in vertebral size^3^,^49^,^50^.

First, we attempted to test our main hypothesis of high lifetime LTPA associating with increased vertebral dimensions. In order to investigate this, we analyzed the association between lifetime LTPA, represented by the LTPA cluster variable, and vertebral CSA. The “inactive” cluster was chosen as the reference category, and the other two clusters were compared to it. Two analyses were conducted (for both genders separately), one with and one without adjustments for vertebral height, BMI, education years and smoking.

We also conducted additional analyses to assess the most important time point in terms of LTPA and increased vertebral dimensions. In order to investigate this, two linear regression analyses were conducted for both genders with the following variables included: 1) LTPA at 14, 31 and 46 years, 2) LTPA at 14, 31 and 46 years, vertebral height, BMI, education years and smoking. The LTPA variables of all three time points were included in every analysis and they thus acted as covariates for each other and their confounding effect was minimized. In the LTPA variables, the category with lowest LTPA frequency was chosen as the reference category, and the other 4 categories were compared to it.

Statistical analyses were conducted using the SPSS software (IBM, Armonk, NY, USA) version 22, 64-bit edition.

## Additional Information

**How to cite this article**: Oura, P. *et al*. Effects of Leisure-Time Physical Activity on Vertebral Dimensions in the Northern Finland Birth Cohort 1966. *Sci. Rep.* **6**, 27844; doi: 10.1038/srep27844 (2016).

## Figures and Tables

**Table 1:** Characteristics of the study sample.

	% (n)	
	Men (N = 524, 44.1%)	Women (N = 664, 55.9%)
Age at imaging, years; mean (SD)	46.8 (0.4)	46.8 (0.4)
CSA of the L4 vertebra, cm^2^; mean (SD)	13.2 (1.7)	10.6 (1.3)
Height of the L4 vertebra, cm; mean (SD)	2.8 (0.1)	2.7 (0.1)
Height, cm; mean (SD)	178.8 (6.1)	165.0 (5.7)
Weight, kg; mean (SD)	85.8 (11.7)	70.5 (13.2)
BMI, mean (SD)	26.9 (3.6)	26.2 (5.0)
BMI, classified (WHO)		
<18.5	0.2 (1)	0.8 (5)
18.5–24.9	33.6 (176)	48.6 (323)
25.0–29.9	48.7 (255)	30.4 (202)
≥30.0	17.6 (92)	20.2 (134)
Education, years		
≤9	3.1 (16)	2.3 (15)
9–12	72.1 (378)	72.7 (483)
>12	24.8 (130)	25.0 (166)
Smoking		
Non-smoker	51.5 (270)	61.0 (405)
Former smoker	33.6 (176)	23.5 (156)
Current smoker	14.9 (78)	15.5 (103)
LTPA at 14 years, participation frequency		
Every other week or less	14.5 (76)	26.8 (178)
Once a week	10.7 (56)	20.6 (137)
Twice a week	19.5 (102)	25.2 (167)
Every other day	31.1 (163)	15.4 (102)
Every day	24.2 (127)	12.0 (80)
LTPA at 31 years, participation frequency		
Once a month or less	21.2 (111)	20.0 (133)
2–3 times/month	12.0 (63)	12.0 (80)
Once a week	20.6 (108)	26.7 (177)
2–3 times/week	31.3 (164)	28.8 (191)
≥4 times/week	14.9 (78)	12.5 (83)
LTPA at 46 years, participation frequency		
Once a month or less	13.9 (73)	11.6 (77)
2–3 times/month	13.0 (68)	11.3 (75)
Once a week	23.1 (121)	19.0 (126)
2–3 times/week	34.2 (179)	41.6 (276)
≥4 times/week	15.8 (83)	16.6 (110)
Lifetime LTPA, clusters		
Inactive	29.4 (154)	29.7 (197)
Moderately active	42.0 (220)	46.4 (308)
Active	28.6 (150)	23.9 (159)

CSA = Cross-sectional area, SD = Standard deviation, BMI = Body mass index, WHO = World Health Organization, LTPA = Leisure-time physical activity.

**Table 2:** Characteristics of the vertebral dimension variables and LTPA variables, presented by cluster.

	% (n)					
	Men			Women		
	Inactive N = 154, 29.4%	Moderately active N = 220, 42.0%	Active N = 150, 28.6%	Inactive N = 197, 29.7%	Moderately active N = 308, 46.4%	Active N = 159, 23.9%
CSA of the L4 vertebra, cm^2^; mean (SD)	13.3 (1.6)	13.2 (1.6)	13.3 (1.8)	10.5 (1.3)	10.5 (1.3)	10.8 (1.2)
Height of the L4 vertebra, cm; mean (SD)	2.8 (0.2)	2.8 (0.1)	2.8 (0.2)	2.7 (0.1)	2.7 (0.1)	2.7 (0.2)
LTPA at 14 years, participation frequency						
Every other week or less	25.3 (39)	13.6 (30)	4.7 (7)	39.1 (77)	24.4 (75)	16.4 (26)
Once a week	16.2 (25)	12.3 (27)	2.7 (4)	24.9 (49)	21.4 (66)	13.8 (22)
Twice a week	18.8 (29)	27.3 (60)	8.7 (13)	18.8 (37)	33.4 (103)	17.0 (27)
Every other day	24.7 (38)	26.8 (59)	44.0 (66)	11.2 (22)	13.0 (40)	25.2 (40)
Every day	14.9 (23)	20.0 (44)	40.0 (60)	6.1 (12)	7.8 (24)	27.7 (44)
LTPA at 31 years, participation frequency						
Once a month or less	72.1 (111)	0.0 (0)	0.0 (0)	67.5 (133)	0.0 (0)	0.0 (0)
2–3 times/month	5.2 (8)	25.0 (55)	0.0 (0)	8.6 (17)	19.5 (60)	1.9 (3)
Once a week	11.0 (17)	39.5 (87)	2.7 (4)	12.7 (25)	47.4 (146)	3.8 (6)
2–3 times/week	3.9 (6)	35.5 (78)	53.3 (80)	5.1 (10)	33.1 (102)	49.7 (79)
≥4 times/week	7.8 (12)	0.0 (0)	44.0 (66)	6.1 (12)	0.0 (0)	44.7 (71)
LTPA at 46 years, participation frequency						
Once a month or less	45.5 (70)	1.4 (3)	0.0 (0)	37.6 (74)	1.0 (3)	0.0 (0)
2–3 times/month	16.2 (25)	18.6 (41)	1.3 (2)	20.8 (41)	11.0 (34)	0.0 (0)
Once a week	13.0 (20)	40.5 (89)	8.0 (12)	18.3 (36)	27.9 (86)	2.5 (4)
2–3 times/week	18.2 (28)	34.1 (75)	50.7 (76)	18.3 (36)	55.8 (172)	42.8 (68)
≥4 times/week	7.1 (11)	5.5 (12)	40.0 (60)	5.1 (10)	4.2 (13)	54.7 (87)

LTPA = Leisure-time physical activity, CSA = Cross-sectional area, SD = Standard deviation.

**Table 3:** The association between lifetime LTPA and vertebral cross-sectional area.

	Men				Women			
	Unadjusted ^1)^		Adjusted ^2)^		Unadjusted ^3)^		Adjusted ^4)^	
	P value	β (95% CI)	P value	β (95% CI)	P value	β (95% CI)	P value	β (95% CI)
Lifetime LTPA, clusters								
Inactive (ref)								
Moderately active	0.775	−0.12 (−0.46; 0.23)	0.673	−0.07 (−0.41; 0.27)	0.730	0.04 (−0.19; 0.27)	0.588	0.06 (−0.17; 0.29)
Active	0.501	0.05 (−0.32; 0.43)	0.460	0.14 (−0.23; 0.52)	0.046*	0.27 (0.004; 0.54)	0.012*	0.34 (0.08; 0.61)

LTPA = Leisure-time physical activity, BMI = Body mass index, β = Beta estimate ([β] = cm^2^), CI = Confidence interval, Ref = Reference category (i.e. to which the other categories were compared), * = p < 0.05.

Characteristics of the models: 1) Adjusted R^2^ = −0.002, F = 0.523, p = 0.593; 2) Adjusted R^2^ = 0.064, F = 5.460, p = 0.009; 3) Adjusted R^2^ = 0.004, F = 2.322, p = 0.099; 4) Adjusted R^2^ = 0.052, F = 5.576, p < 0.001.

Two regression analyses were run for both genders, one with and one without adjustments for BMI, vertebral height, education years and smoking.

**Table 4:** The association between leisure-time physical activity at 14, 31 and 46 years of age and vertebral cross-sectional area.

	Men				Women			
	Unadjusted ^1)^		Adjusted ^2)^		Unadjusted ^3)^		Adjusted ^4)^	
	P value	β (95% CI)	P value	β (95% CI)	P value	β (95% CI)	P value	β (95% CI)
LTPA at 14 years								
Every other week or less (ref)								
Once a week	0.437	−0.23 (−0.81; 0.35)	0.083	−0.50 (−1.07; 0.07)	0.418	−0.12 (−0.40; 0.17)	0.450	−0.11 (−0.39; 0.17)
Twice a week	0.085	−0.44 (−0.95; 0.06)	0.027*	−0.55 (−1.05; −0.06)	0.376	0.12 (−0.15; 0.40)	0.233	0.16 (−0.11; 0.43)
Every other day	0.938	0.02 (−0.45; 0.49)	0.802	−0.06 (−0.52; 0.40)	0.101	0.26 (−0.05; 0.58)	0.080	0.27 (−0.03; 0.58)
Every day	0.394	−0.21 (−0.70; 0.28)	0.162	−0.34 (−0.82; 0.14)	0.367	0.16 (−0.19; 0.50)	0.431	0.14 (−0.20; 0.47)
LTPA at 31 years								
Once a month or less (ref)								
2–3 times/month	0.551	−0.16 (−0.70; 0.37)	0.609	−0.13 (−0.65; 0.38)	0.676	0.08 (−0.28; 0.43)	0.810	0.04 (−0.31; 0.39)
Once a week	0.528	−0.15 (−0.60; 0.31)	0.905	−0.03 (−0.47; 0.42)	0.618	0.07 (−0.22; 0.37)	0.447	0.11 (−0.18; 0.40)
2–3 times/week	0.630	−0.11 (−0.54; 0.32)	0.998	−0.00 (−0.42; 0.42)	0.206	0.19 (−0.11; 0.49)	0.097	0.25 (−0.04; 0.54)
≥4 times/week	0.405	−0.22 (−0.73; 0.30)	0.525	−0.16 (−0.66; 0.34)	0.013*	0.47 (0.10; 0.84)	0.003*	0.55 (0.18; 0.91)
LTPA at 46 years								
Once a month or less (ref)								
2–3 times/month	0.090	0.49 (−0.08; 1.05)	0.145	0.41 (−0.14; 0.96)	0.265	0.23 (−0.18; 0.64)	0.273	0.22 (−0.18; 0.63)
Once a week	0.378	0.23 (−0.28; 0.74)	0.435	0.20 (−0.30; 0.70)	0.262	0.21 (−0.16; 0.58)	0.249	0.21 (−0.15; 0.58)
2–3 times/week	0.394	0.21 (−0.28; 0.74)	0.406	0.20 (−0.27; 0.68)	0.624	0.08 (−0.25; 0.42)	0.708	0.06 (−0.28; 0.40)
≥4 times/week	0.234	0.34 (−0.22; 0.90)	0.216	0.35 (−0.21; 0.91)	0.521	0.13 (−0.27; 0.53)	0.481	0.14 (−0.25; 0.54)

BMI = Body mass index, LTPA = Leisure-time physical activity, β = Beta estimate ([β] = cm^2^), CI = Confidence interval, Ref = Reference category (i.e. to which the other categories were compared), * = p < 0.05.

Characteristics of the models: 1) Adjusted R^2^ = −0.005, F = 0.777, p = 0.674; 2) Adjusted R^2^ = 0.066, F = 3.044, p < 0.001; 3) Adjusted R^2^ = 0.008, F = 1.464, p = 0.133; 4) Adjusted R^2^ = 0.060, F = 3.343, p < 0.001.

Two regression analyses were run for both genders, one with and one without adjustments for BMI, vertebral height, education years and smoking. LTPA variables were adjusted for each other.
